# Intrarenal parapelvic cysts presenting as allograft dysfunction 10 years post-renal transplantation: a case report

**DOI:** 10.1016/j.eucr.2025.103304

**Published:** 2025-12-03

**Authors:** Hyunwoong Harry Chae, Terri Ser, David Harriman, Christopher Nguan

**Affiliations:** a Faculty of Medicine, University of British Columbia, Vancouver, BC, Canada; b Department of Urologic Sciences, University of British Columbia, Vancouver, BC, Canada

## Abstract

Parapelvic cysts (PPCs) are rare renal cysts that can masquerade as ureteric obstruction and hydronephrosis on imaging. We describe an atypical case of intrarenal PPCs in a renal allograft that presented 10 years post-transplantation with graft dysfunction and imaging features mimicking hydronephrosis, thereby creating a diagnostic pitfall. To avoid unnecessary investigations and interventions in these patients, transplant professionals must consider this diagnosis early in their differential when managing such presentations.

## 1 Introduction

Parapelvic cysts (PPCs) are rare renal cysts that originate adjacent to the renal sinus, in contrast to simple renal cysts, which typically arise from the peripheral renal cortex.^1^ They are often asymptomatic and require no treatment, and are not associated with cancer. We hereby report an atypical case of PPCs, presenting as the cause of allograft dysfunction and mimicking hydronephrosis on imaging, in a patient 10-year post-renal transplant.

## 2 Case report

**Fig. 1:**
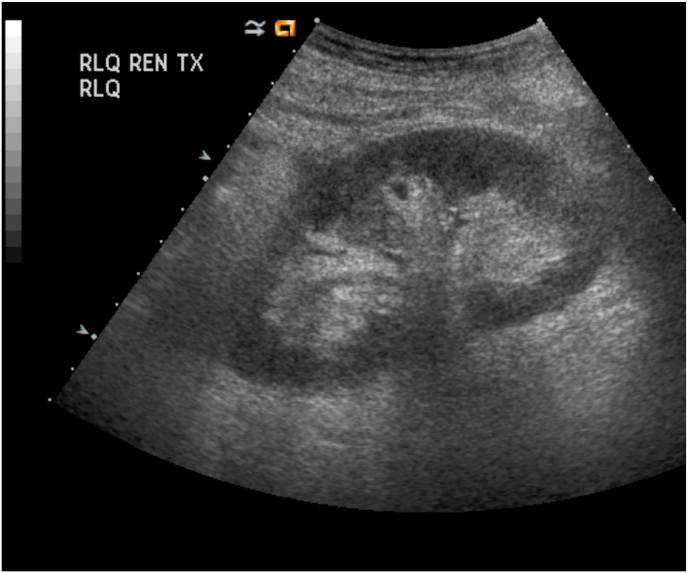
First post-transplant ultrasound showing normal graft kidney. (June 2014).

A 71-year-old female who received a living-related donor renal transplant 10 years previously presented to the clinic in January 2024 with new allograft dysfunction. The cause of her end stage renal disease (ESRD) was hypertension. Her kidney transplant surgery (May 2014) was uncomplicated with normal post-transplant ultrasound (US) findings (Fig. 1; June 2014). She had done well in the 10-year interval following, with a stable baseline creatinine level of 85 μmol/L and no episodes of rejection.

**Fig. 2:**
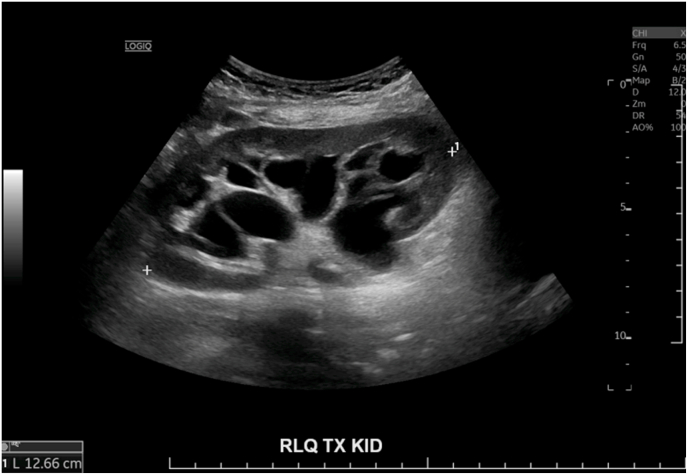
Ultrasound of allograft kidney mimicking hydronephrosis. (July 2024).

**Fig. 3:**
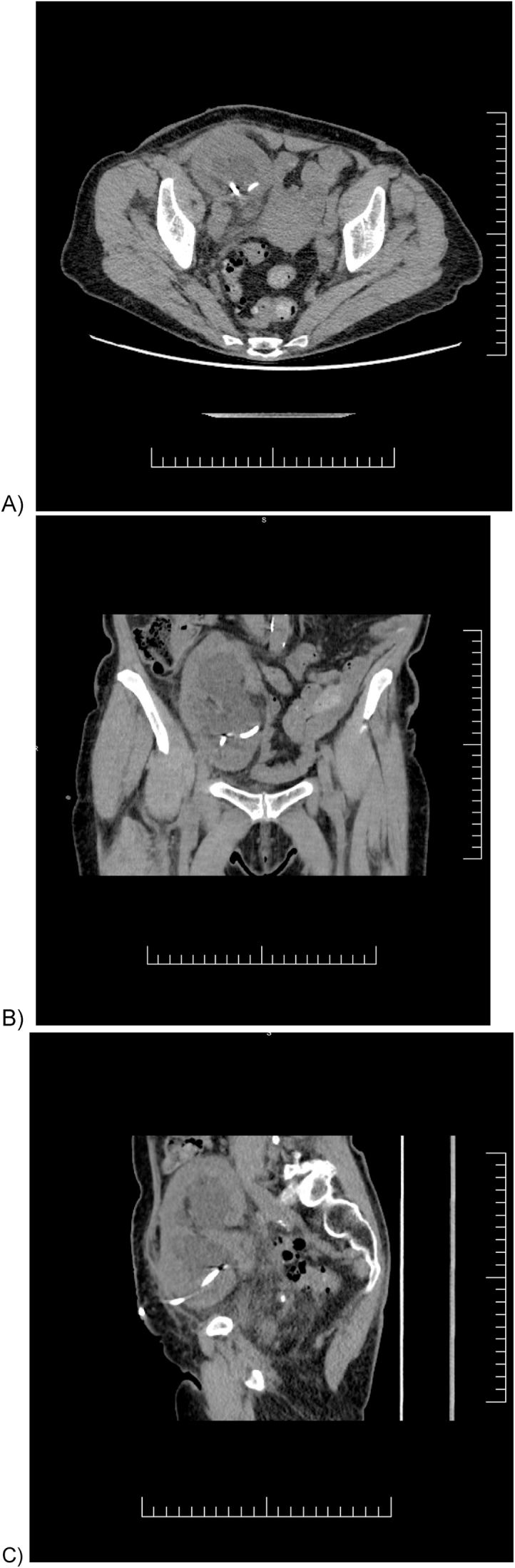
Non-contrast CT pelvis showing allograft kidney with possible graft hydronephrosis and nephrostomy tube (September 2024). A) axial, B) coronal, C) sagittal views.

**Fig. 4:**
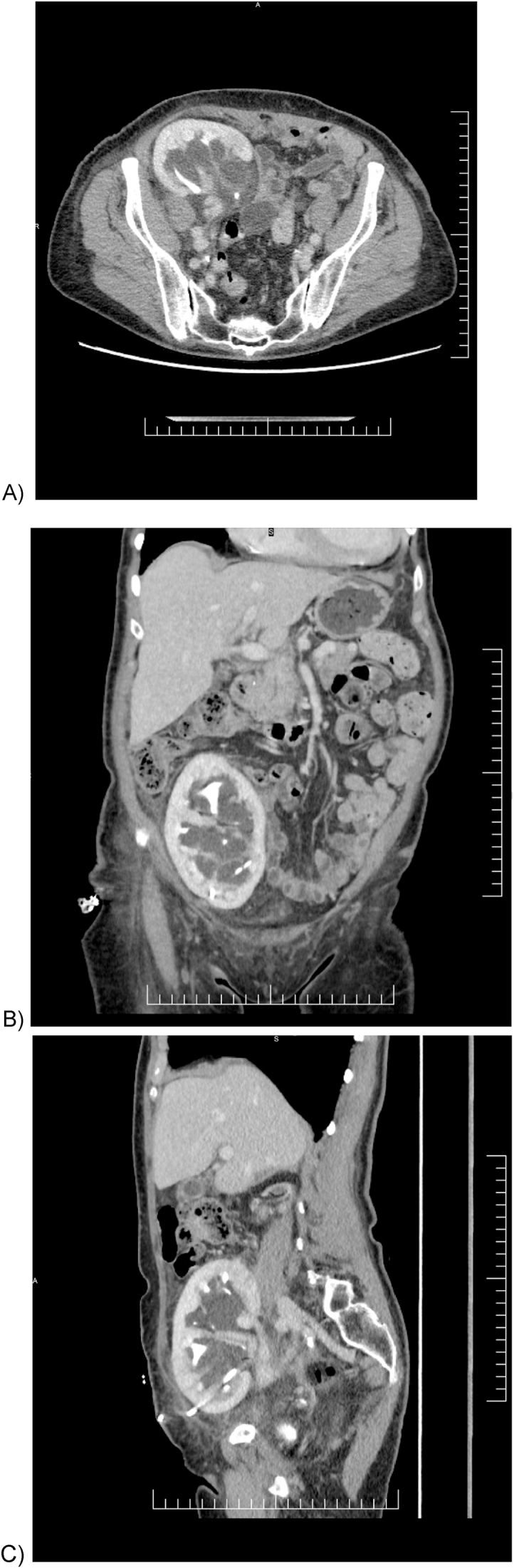
Contrast-enhanced computed tomography intravenous pyelogram (CT IVP) demonstrating the renal allograft with multiple parapelvic cysts, correctly placed nephrostomy tube, and persistence of graft “hydronephrosis”. (October 2024). A) axial, B) coronal, C) sagittal views.

Her presenting illness was characterized by moderate allograft dysfunction with an elevated creatinine of 120 μmol/L in January 2024 detected on routine bloodwork, with US demonstrating moderate hydronephrosis with no other cause identified. In July 2024, her creatinine had increased to 122 μmol/L, and a repeat US (Fig. 2) revealed severe hydronephrosis, which persisted even after voiding. Cystoscopy done in August 2024 revealed a normal bladder without evidence of pathology to account for presumed obstruction. Following this, a percutaneous nephrostomy tube (NT) was placed to decompress the allograft (August 2024). The NT initially drained 250 cc/day but then tailed off to drain minimal amounts in the days following. A follow-up non-contrast CT scan (September 2024; Fig. 3) continued to show severe hydronephrosis despite correct positioning of the NT. A contrast CT scan (October 2024; Fig. 4) demonstrated correct NT placement, multiple PPCs within the interior of the kidney, and decompressed renal pelvis and calyces, despite minimal output from the NT.

In November 2024, US-guided aspiration of the PPCs was performed. Fluid cytology showed acellular proteinaceous material with very rare chronic inflammatory cells. No malignant cells were identified, and the culture was negative. No progressive hydronephrosis was noted on this study. Clamp trial of NT demonstrated no further hydronephrosis on US, and the NT was removed. Her creatinine at this time was 110 μmol/L, and she was having normal urine output. As such, it was deemed that the patient was not functionally obstructed from the PPCs, and she was discharged to routine follow-up.

**Fig. 5:**
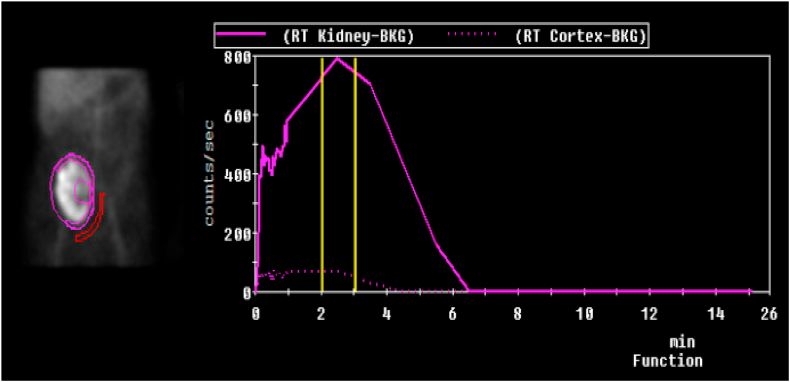
Lasix nuclear renogram demonstrating no obstruction. (September 2025).

In September 2025, a follow-up Lasix renogram was performed to assess allograft drainage and function, which demonstrated unobstructed outflow (Fig. 5). Serum creatinine at this time was 114 μmol/L.

## 3 Discussion

This case highlights a diagnostic dilemma due to the presence of multiple PPCs interior to the kidney, mimicking hydronephrosis and threatening kidney outflow obstruction. There is limited literature regarding PPCs, and as such, the underlying pathophysiology is unclear. Some reports suggest an origin from lymphatic obstruction,^2^ while others suggest they are an embryological remnant arising from the mesonephric duct.^3^^,^^4^ One report described that PPCs consist of one flat layer of epithelial cells, suggesting a different origin from the urinary tract epithelium, which is composed of transitional cells.^5^^,^^6^ It remains uncertain in kidney transplant scenarios whether PPCs that develop in allografts originate from donor-derived elements or arise secondary to recipient-related processes. Presentation of PPCs is also poorly understood in terms of evolution in renal allografts over time. In our case, multiple, large PPCs appeared 10 years post-transplant, likely having been present for some time beforehand, growing slowly and causing no discernible issues. These uncertainties complicate potential approaches to screening (i.e., assessing risk for cyst development prior to graft donation), prevention, and management. Further studies are needed to delineate the underlying pathophysiology of PPCs, especially in the context of transplantation.

While PPCs are rare, previous case reports have described that they can present similarly to hydronephrosis on imaging.^7^, ^8^, ^9^ Despite this, due to a lack of studies and limited awareness of this renal pathology, they are commonly initially misdiagnosed as hydronephrosis, and there are currently no clear diagnostic workflow or management guidelines for this condition, especially in the context of renal transplant patients with a solitary functioning kidney.

This case created a challenging diagnostic dilemma for several reasons. First, the patient maintained stable graft function for 10 years post-transplant, making the emergence of a new structural abnormality at this late stage unexpected. Second, while most simple renal cysts are spherical in shape and located in the renal cortex, our case showed multiple, irregularly shaped cysts residing within the sinus, which is an atypical imaging finding that initially raised concern for conditions other than cysts, including benign and malignant tumors.^10^ Third, the PPCs occurred in an allograft kidney, compared to a native kidney, which added complexity and urgency to the diagnostic workup in terms of ensuring adequate decompression in the short term and ongoing management in the long term. Transplant recipients are at higher risk of certain complications due to factors such as surgical manipulation, immunosuppression, and immune-mediated responses of the graft. As such, graft rejection, lymphoceles, ureteral strictures, malignancy, infection, medication toxicities, and recurrence of underlying renal disease must be given greater consideration in transplant recipients presenting with rising creatinine and apparent hydronephrosis. These transplant-specific considerations significantly broaden the differential diagnosis compared to patients with native kidneys.^11^, ^12^, ^13^

In our case, the atypical presentation of multiple large and nonuniform parapelvic cysts abutting the renal sinus interior to the renal allograft masquerading as ureteric obstruction and graft hydronephrosis resulted in multiple investigations, which ultimately turned out to be negative with confirmation of a natively unobstructed upper tract collecting system of the allograft kidney. The cause of the incident rise in serum creatinine at initial presentation was not well understood but appeared to be coincidental to the imaging findings, and graft function eventually returned to baseline during investigations.

## 4 Conclusion

In summary, we described a rare case of intrarenal PPCs in an allograft kidney presenting as graft dysfunction and mimicking hydronephrosis on imaging. Our report highlights the importance of increasing awareness of PPCs among transplant professionals. When a kidney transplant recipient presents with hydronephrosis and unexplained graft dysfunction accompanied by similar imaging findings to this case, PPCs must be considered in the differential early in order to reach the correct diagnosis and avoid potentially unnecessary investigations or interventions.

## CRediT authorship contribution statement

**Hyunwoong Harry Chae:** Conceptualization, Investigation, Methodology, Writing – original draft, Writing – review & editing. **Terri Ser:** Writing – original draft, Writing – review & editing. **David Harriman:** Writing – review & editing. **Christopher Nguan:** Conceptualization, Supervision, Writing – original draft, Writing – review & editing.

## Funding

This research did not receive any specific grant from funding agencies in the public, commercial, or not-for-profit sectors.

## Declaration of competing interest

The authors declare that they have no competing financial interests or personal relationships that could have influenced the work reported in this paper.
